# Generation of few-cycle multi-millijoule 2.5 *μ*m pulses from a single-stage Cr^2+^:ZnSe amplifier

**DOI:** 10.1038/s41598-020-64330-8

**Published:** 2020-05-08

**Authors:** Yi Wu, Fangjie Zhou, Esben W. Larsen, Fengjiang Zhuang, Yanchun Yin, Zenghu Chang

**Affiliations:** 1 CREOL and Department of Physics, University of Central Florida, Orlando, Florida 32816 USA; 2 Quantum Optics and Laser Science Group, Blackett Laboratory, Imperial College London, London, SW7 2BW UK

**Keywords:** Ultrafast photonics, Nonlinear optics

## Abstract

Lasers capable of generating attosecond X-ray pulses in the water window (282 to 533 eV) through high-order harmonic generation are normally based on inefficient, multi-stage optical parametric amplifiers or optical parametric chirped pulse amplifiers pumped by femtosecond or picosecond lasers. Here we report a very efficient single amplification stage laser based on traditional chirped pulse amplification capable of producing 4 mJ, near-transform limited 44 fs (<6 cycles), 1 kHz pulses centered at 2.5 *μ*m. The ≈90 GW peak power is the highest value ever reached at this wavelength. In order to fully compress the laser pulses our system is built in a nitrogen box. Our system utilizes water cooled chromium doped zinc selenide (Cr^2+^:ZnSe) as the gain medium and is pumped by a commercial nanosecond holmium doped yttrium-aluminum-garnet (Ho:YAG) laser.

## Introduction

The natural time scale of electron dynamics in atoms, molecules, and condensed matter is on the order of attoseconds. Since the first demonstration of tabletop attosecond light sources from high-order harmonic generation (HHG) in 2001^1,2^, titanium doped sapphire (Ti:Sapphire) lasers centered at 800 nm have been the workhorse for attosecond research. These systems have enabled coherent pulses covering the extreme ultraviolet spectral range of 15–150 eV^3,4^. However, for many applications in ultrafast physics and chemistry it is instrumental to extend attosecond capabilities into and beyond the soft X-ray (SXR) “water window”, i.e., ≥530 eV. High-energy attosecond SXR pulses would enable time-resolved studies of core-level transitions in organic and biological systems containing carbon, nitrogen, oxygen, and several other key elements.

In the past two decades many methods of extending the photon energy have been suggested, such as producing HHG in ions or from core level electrons^5,6^. However, in 2001 it was experimentally demonstrated that a more straightforward method of extending the cutoff frequency was merely to increase the driving laser wavelength, since the electron quiver energy, and thereby the photon cutoff energy, scales quadratically with the driving wavelength^7^. In the initial work this was performed with a home-built 100 *μ*J optical parametric amplifier (OPA) pumped by a few mJ femtosecond Ti:Sapphire laser that was based on traditional chirped pulse amplification (CPA) technology^8^. The low pulse energy of the OPA meant that the HHG cut-off energy was limited to approximately 150 eV for a driving wavelength of 1500 nm, while the cutoff was limited to roughly 40 eV for 800 nm pulses with the same pulse energy.

In recent years, much higher energy (1–2 mJ) OPAs typically centered at 1800 nm have become commercially available. These systems typically have pulse durations around 40 fs, but by deploying pulse compression techniques such as gas-filled hollow-core fibers followed by fused silica wedges pulses with >0.5 mJ and durations less than two-cycles can be produced. This has enabled photon energies covering the SXR water window^9–11^. An alternative method of generating high-energy short wave infrared pulses based on optical parametric CPA (OPCPA) have led to carrier-envelope phase stable pulses at 1.7 *μ*m with pulse energies up to 3 mJ at 1 kHz ^12,13^. By generating HHG with these two types of novel systems it has been possible to generate soft x-ray pulses with durations around 50 as^14,15^. Very recently, attosecond transient absorption spectroscopy at the Nitrogen K-edge (400 eV) and Titanium L-edge (460 eV) has been demonstrated with both OPAs and OPCPAs^16,17^.

Nevertheless, it is crucial to further extend both the HHG cutoff photon energy and flux in order to enable studies of more complicated systems. In order to significantly improve this we need to improve the driving laser systems. As an example we will review here the three-stage OPCPA in reference^13^. This system is centered at 1.7 *μ*m and is pumped by a 20 mJ, 4 ps, three-stage Ti:Sapphire CPA. The power amplifiers of the CPA are pumped by 527 nm nanosecond pulses with a total energy of more than 100 mJ supplied by Q-switched Nd:YLF lasers. The energy conversion efficiency of the 527 nm pump lasers to the 1.7 *μ*m output is low, <3%, which means that the upkeep cost is high for this laser. Moreover, the multi-stage configuration of both the Ti:Sapphire laser and the OPCPA makes it difficult to operate on a day-to-day basis.

Research has indicated that lasers capable of generating isolated attosecond pulses in the water window and beyond must meet the following requirements^9–11,18^: (1) center wavelength >1.5 *μ*m, (2) pulse energy >0.25 mJ, and (3) pulse duration <2 cycles. Recent progress in mid-infrared laser technology^19^ suggests alternative laser configurations that can potentially meet these requirements. In particular, CPAs based on the four-level gain medium Cr^2+^:ZnSe seem to be a promising choice^20–22^. The broad vibronic emission spectrum of Cr^2+^:ZnSe is from 2 *μ*m to 3.3 *μ*m, which means that it would in principle be possible to build 3–4 cycle lasers based solely on Cr^2+^:ZnSe. Furthermore, Cr^2+^:ZnSe can be pumped using Ho:YAG lasers at 2.09 *μ*m therefore the quantum efficiency can be as high as 80% for a Cr^2+^:ZnSe centered at 2.5 *μ*m. Moreover, this gain material can be used to develop multi-kHz laser systems.

We have recently reported on a three-stage chirped pulse amplifier utilizing this gain crystal with an output of 2.3 mJ and a pulse duration of 88 fs^21^. In this paper, we demonstrate a CPA system that can produce 4 mJ, 44 fs, 1 kHz pulses in a single-stage amplifier.

## Results

**Figure 1:**
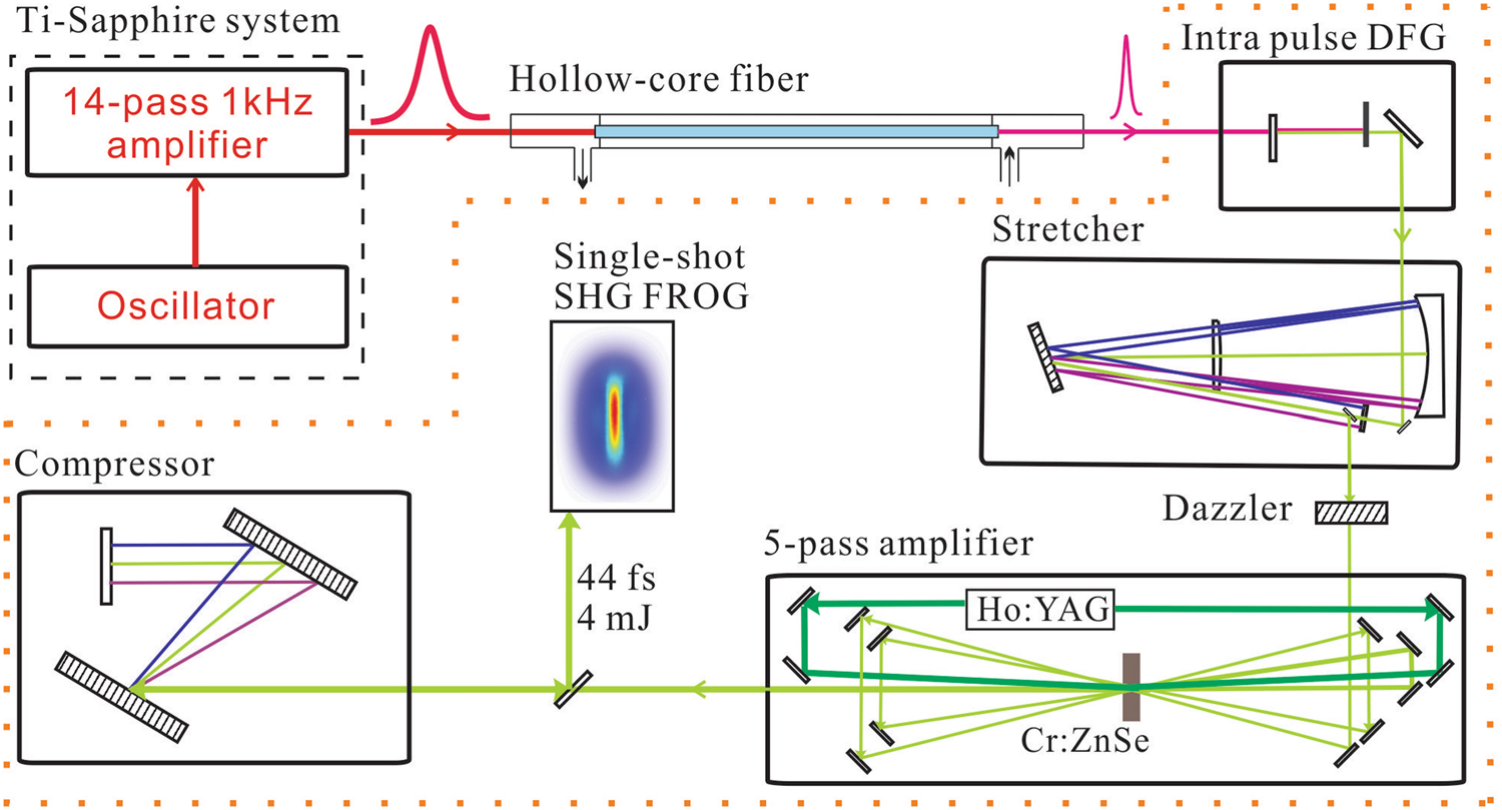
Layout of Cr^2+^:ZnSe CPA system.

The layout of the Cr^2+^:ZnSe laser system is shown in Fig. 1. The seed pulses for the system are generated the same way as in the previously reported three-stage amplifier and is therefore only outlined here. A 14-pass Ti:Sapphire CPA system is used to generate 1.5 mJ, 30 fs, 800 nm pulses at a repetition rate of 1 kHz. These pulses are spectrally broadened and compressed in a conventional argon-filled hollow-core fiber setup followed by a set of double-angle chirped mirrors. The output beam is focused into a 0.8 mm thick BIBO crystal cut for type-I phase matching that allows for intrapulse difference frequency generation (IDFG). The idler of the IDFG spectrum spans from 1.8 *μ*m to 4.2 *μ*m, while the signal spans from 1 *μ*m to 1.8 *μ*m^23^. Since the peak of the emission wavelength of Cr^2+^:ZnSe is around 2.5 *μ*m we use the idler to seed the laser chain. A long pass filter, which blocks wavelengths shorter than 2 *μ*m, is used to isolate and optimize the seed pulse. The pulse energy after the filter is approximately 3 *μ*J.

**Figure 2:**
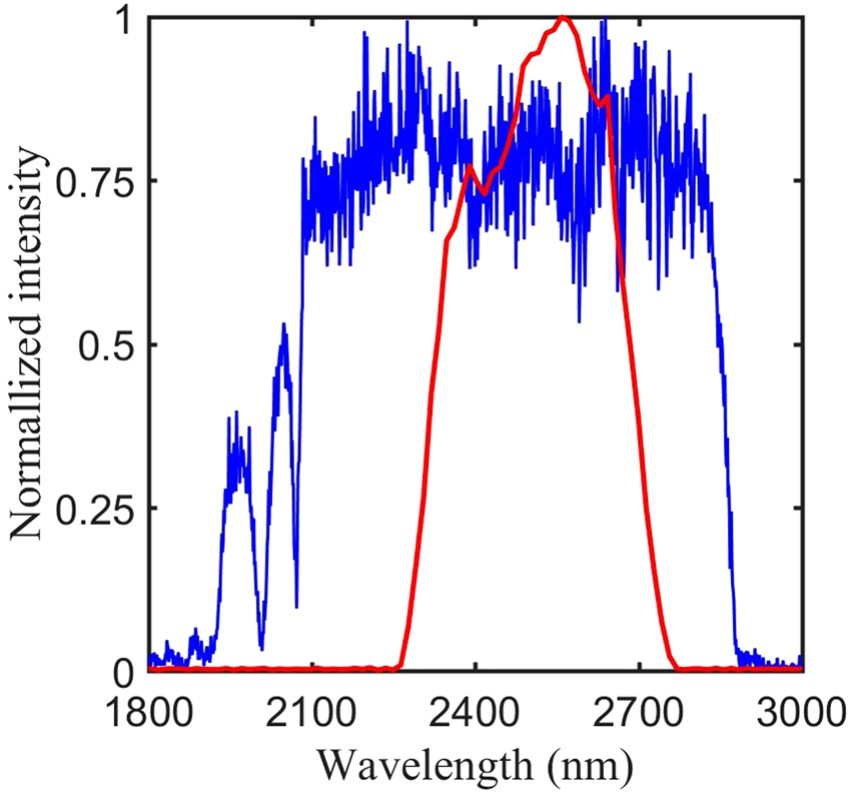
Normalized seed (blue) and amplifier (red) spectra of the laser system. Due to the low energy the seed is measured using a scanning spectrometer waveScan MIR from APE, while the amplified spectrum is measured using a SM301 spectrometer from Spectral Products.

The seed is stretched in a double-pass aberration free Öffner-triplet stretcher containing a reflective grating with groove density of 300 lines/mm. The radius of curvature of the convex and the concave mirror of the Öffner telescope is 1 m and 0.5 m, respectively. The stretcher uses an incidence angle slightly larger than the Littrow configuration to maximize the efficiency of the gratings, while allowing us to experimentally separate the input and output beams. The stretched pulse duration is approximately 300 ps. Due to the low pulse energy and average power (<2 *μ*J and <2 mW) after the stretcher we were unable to directly measure the seed energy after the stretcher. Nevertheless, we measured the stretcher efficiency to ~30% using a separate broadband source, so we estimate that after the stretcher the seed beam has a pulse energy around 1 *μ*J. A mid-infrared acousto-optic programmable dispersive filter, DAZZLER^24^, is deployed after the stretcher to pre-compensate high-order phase in the amplifier as detailed below. The DAZZLER further reduces the seed pulse energy. After the DAZZLER the pulses are directed into a five-pass amplifier. The gain medium is a 40 mm long rectangular poly-crystalline Cr^2+^:ZnSe crystal (IPG Photonics) with a height of 5 mm and a width of 10 mm. The crystal is water cooled to 18 °C, and is pumped by a Q-switched 25 mJ, 50 ns, 2.09 *μ*m Ho:YAG laser (IPG Photonics) from both ends. The diameter of the pump beam is 3.2 mm and 75% of the pump energy is absorbed, The seed diameter is ~2 mm. After the first pass the pulse energy remains too low to measure (<2 *μ*J). However, the energy is boosted to 25 *μ*J and 350 *μ*J in the second and third pass, respectively. The amplification factor is therefore around 14 in the third pass. The gain is decreased in the fourth pass resulting in an energy of 1.8 mJ, representing an amplification factor around 5. After the fifth pass the gain is further saturated with an output energy of 5.9 mJ. Between each pass a lens is placed so that the seed beam is focused before reaching the crystal in order to balance the thermal lensing inside the crystal^25^. The seed and amplified spectra is presented in Fig. 2. The single-stage configuration thus achieves a similar output energy to the recently reported 3-stage Cr^2+^:ZnSe amplifier. During the amplification process the long-pass filter in front of the stretcher is removed, since the both the signal and the pump beam from the IDFG process are outside the vibronic emission spectrum for Cr^2+^:ZnSe, and thus do not affect the amplification. This ensures a slightly higher seed pulse for the system, and avoids adding unwanted spectral phase onto the seed pulse. After the amplifier the pulses are compressed in a double-pass grating compressor with same groove density as the stretcher. The throughput of the compressor is 68% resulting in an output pulse energy of 4 mJ. Finally, the temporal profile of the output was characterized by a home-built single-shot second harmonic Frequency Resolved Optical Gating (FROG)^26^. The entire system is built inside a sealed box, and is purged with nitrogen during operation. The purging is needed, due to strong absorption in water vapor for wavelengths ≥2.5 *μ*m.

### Thermal lensing in Cr^2+^:ZnSe

**Figure 3:**
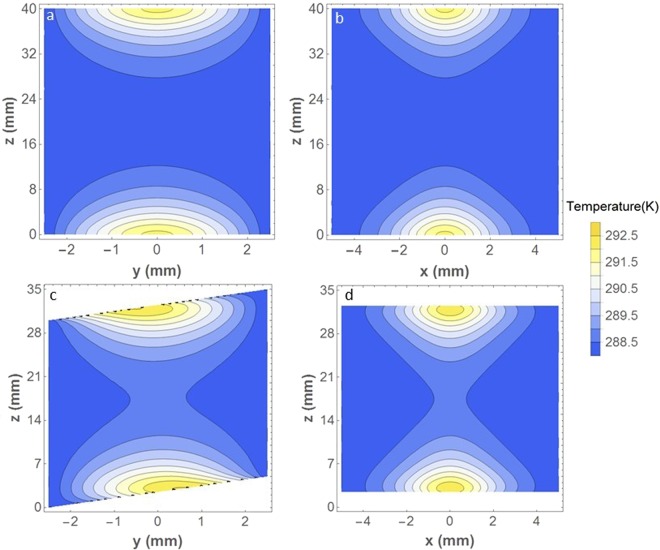
Comparison of thermal distribution for crystal for the parameters given in Table 1 cut for normal incidence (**a**,**b**) and Brewster cut crystal (**c**,**d**). In all the subfigures x, y and z are the horizontal, vertical and propagation directions, respectively.

In order to evaluate the effects of thermal lensing of the amplifier we performed simulations using the LAS-CAD software package (LAS-CAD GmbH). Our simulations consisted of finite element analysis of the thermal distribution and lensing of the crystal. The material parameters used for our simulations are listed in Table 1, where $\kappa$ is thermal conductivity, $w$ is the pump beam radius, *P* is the absorbed average pump power, $\eta$ is the quantum efficiency given by the ratio between the absorption and emission photon wavelengths, $\alpha$ is the linear thermal expansion coefficient, $n$ is the refractive index at the seed wavelength of 2500 nm, *dn*/*dT* is the temperature dependence of the refractive index at this wavelength, $Y$ is Young’s Modulus, and finally $\nu$ is Poisson’s ratio. In our simulations we compared Brewster cut crystals with plane incidence crystals. Figure 3(a,b) show the simulated temperature distributions for the normal incidence crystal, while (c) and (d) show this for the Brewster’s cut crystal. In the figures the x, y, and z axis represent the horizontal, vertical, and propagation directions of the various crystals, respectively. For the normal incidence crystal a minor level of asymmetry between horizontal and vertical axis is observed. Using the software package, we were able to estimate the focal length of the normal incidence crystal to be 1.22 m in the x-z plane and 0.95 m in the y-z plane. The Brewster cut crystal clearly exhibits a higher level of temperature asymmetry between the vertical and horizontal direction. This asymmetry leads to an enhanced amount of astigmatism and other aberrations of the amplified beam. Unfortunately, the software was unable to estimate the aberrations caused by thermal lensing in the Brewster cut crystal, due to the severity of the asymmetry of the temperature distribution. Nevertheless, it is clear that the aberrations will be significantly worse than for the normal incidence case as the peak temperature difference is the same in either case, while the distribution is more symmetric for the normal incidence crystals. In comparison with stage one of reference^21^ the beam size of the pump was increased by a factor ≈2.7, while the pulse energy was increased by less than a factor 2, meaning that the fluence is more than halved here, thus further reducing the effects of thermal lensing. Finally, the double-sided pumping leads to a more homogeneous gain profile along the crystal.

**Table 1:** Material properties of Zinc Selenide^33,34^.

*κ* [W/(W · m)]	*w*[*mm*]	*P*[*W*]	*η*[%]	*α* [K^−1^]	n	*dn*/*dT* [K^−1^]	*Y* [GPa]	*ν*
18	1.3	20	83.6	7.3 · 10^−6^	2.44	7 · 10^−5^	75.8	0.28

### Dispersion and compression of the system

**Figure 4:**
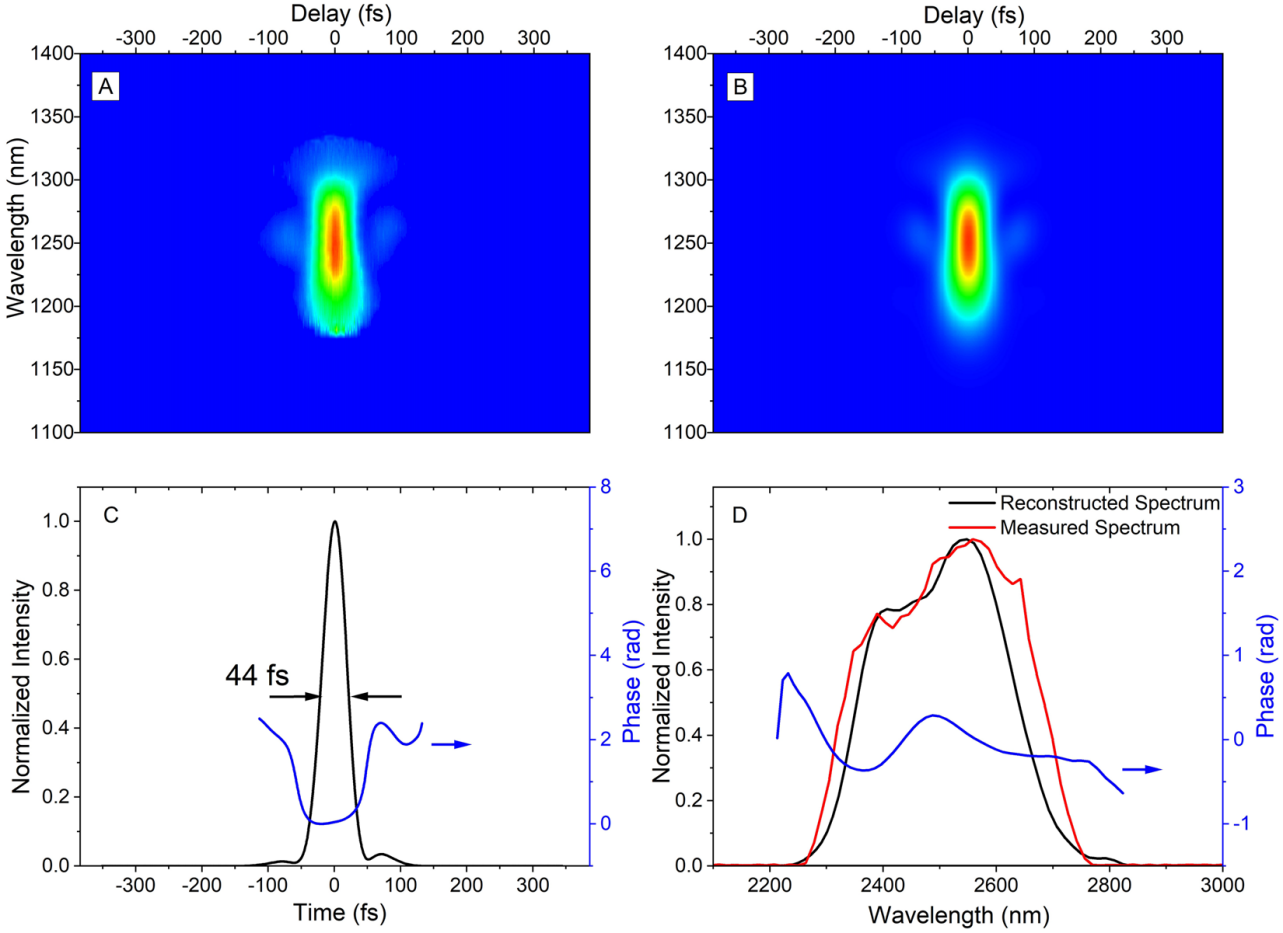
FROG measurement and retrieval. (**A**) Measured FROG trace. (**B**) Retrieved FROG trace. (**C**) Black curve, retrieved pulse intensity profile; blue curve, retrieved temporal phase. (**D**) Red curve, measured spectrum with SM301-EX (Spectral Product, LLC); black curve, retrieved spectrum; Blue curve, retrieved spectral phase.

The output spectrum of the laser can be seen in Fig. 4(D). The bandwidth supports pulses down to 43 fs. In order to compress the pulses we calculated the dispersion of the stretcher up to the fourth order for an incidence angle of 25 degrees. We also calculated the effects of material dispersion in the amplifier chain up to the fourth order based on the Sellmeier equations for CaF_2_ and ZnSe^27,28^, The calculated group delay dispersion (GDD), the third order dispersion (TOD) and the fourth order dispersion (FOD) for the material and stretcher are given in Table 2.

**Table 2:** Dispersion calculations for 20 mm of CaF2 and 160 mm ZnSe and the Öffner stretcher at an incidence angle of 25 degrees and a grating separation of 20 cm.

Optical element	GDD (fs^2^)	TOD (fs^3^)	FOD (fs^4^)
Stretcher	1.18 · 10^6^	−5.99 · 10^6^	5.31 · 10^7^
Material	−3.25 · 10^4^	6.67 · 10^4^	9.2 · 10^3^

The system has a large amount of material dispersion due to the long gain crystal. We numerically optimized the grating angle and separation of our grating compressor to compensate the combined GDD and TOD of the stretcher and amplifier. We found that it was possible to compensate this GDD and TOD with a simple double-pass two-grating compressor with the same groove density as the stretcher. However, by doing so the system was left with a FOD of −7 · 10^6^ fs^4^, which would limit the pulse duration to approximately 175 fs. In principle, shorter pulses could be obtained by tweaking the grating angle and separation and obtain a compromise between GDD, TOD and FOD that would lead to a shorter pulse - but not transform limited pulse. Without the DAZZLER we were able to generate around 70 fs pulses. We therefore chose to deploy the mid-infrared DAZZLER to be able to pre-compensate high-order chirp. The measurements were conducted when the laser enclosure was purged with dry nitrogen and the humidity is <5% (This humidity is limited by our detector). The results are shown in Fig. 4. The FWHM pulse duration is 44 fs, which is the shortest pulse achieved for mJ lasers centered at 2.5 *μ*m. Experimentally we found that the pulse duration was optimized by adding a FOD of approximately 3.5 · 10^6^ fs^4^. We attribute the minor differences in experimental values and theoretical values to imprecision in grating angles and separations, the intrinsic dispersion of the DAZZLER, which is not included in our calculations, and residual water vapor after the purging.

## Discussion

In summary, we have demonstrated a single-stage Cr^2+^:ZnSe chirped pulse amplifier capable of producing 4 mJ, 44 fs laser centered at 2.5 *μ*m. The optical-to-optical conversion efficiency from the 2.09 *μ*m pump to the 2.5 *μ*m laser is ≈16%, which is much higher than our previously reported OPCPA (<3%)^13^ and other state-of-the-art OPCPAs (<10%)^18,29^. The wall-plug efficiency is even higher for the Cr^2+^:ZnSe laser since the power supply of the Ho:YAG laser consumes 2.1 kW of which 0.8 kW is for the chillers whereas the three Nd:YLF lasers for the OPCPA require 5.7 kW to operate (power for chillers not included). The output energy can be further increased by adding additional amplification stages. The peak power, 90 GW, is the highest achieved at 2.5 *μ*m. By purging the entire laser system of water vapor with a nitrogen atmosphere and deploying a mid-infrared DAZZLER we were able to halve the pulse duration, compared to our previous system^21^, and produce near transform-limited pulses of 44 fs corresponding to less than 6 optical cycles. Further self-compression to less than two cycles in either a solid medium with negative GVD materials^30^ or through hollow-core fiber compression^9^ in order to generate isolated attosecond X-rays in the SXR water window seems very feasible. The carrier-envelope phase (CEP) of the seed pulses to our laser may be stable since they are produced through IDFG. Therefore, stabilizing the CEP of the entire system should be possible by compensating for the slow phase drift introduced by the grating based pulse stretcher and compressor using techniques previously developed for Ti:Sapphire lasers^31,32^.

## Data Availability

Data are available upon reasonable requests to the corresponding author.
